# Occurrence of Aflatoxin M1 in Cow, Goat, Buffalo, Camel, and Yak Milk in China in 2016

**DOI:** 10.3390/toxins14120870

**Published:** 2022-12-10

**Authors:** Nan Zheng, Li Min, Dagang Li, Sheng Tan, Yanan Gao, Jiaqi Wang

**Affiliations:** 1 Key Laboratory of Quality & Safety Control for Milk and Dairy Products of Ministry of Agriculture and Rural Affairs, Institute of Animal Sciences, Chinese Academy of Agricultural Sciences, Beijing 100193, China; 2 Ministry of Agriculture Key Laboratory of Animal Nutrition and Feed Science in South China, Guangdong Public Laboratory of Animal Breeding and Nutrition, Institute of Animal Science, Guangdong Academy of Agricultural Sciences, Guangzhou 510640, China

**Keywords:** occurrence, aflatoxin M1, raw milk, five dairy species, China

## Abstract

In this present study, 195 cow milk, 100 goat milk, 50 buffalo milk, 50 camel milk, and 50 yak milk samples were collected in China in May and October 2016. The presence of aflatoxin M1 (AFM1) was determined using enzyme-linked immunosorbent assay method. For all cow milk samples, 128 samples (65.7%) contained AFM1 in concentrations ranging from 0.005 to 0.191 µg/L, and 6 samples (3.1%) from Sichuan province in October were contaminated with AFM1 above 0.05 µg/L (EU limit). For all goat milk samples, 76.0% of samples contained AFM1 in concentrations ranging from 0.005 to 0.135 µg/L, and 9 samples (9.0%) from Shanxi province in October were contaminated with AFM1 above 0.05 µg/L. For all buffalo milk samples, 24 samples (48.0%) contained AFM1 in concentrations ranging from 0.005 to 0.089 µg/L, and 2 samples collected in October were contaminated with AFM1 above 0.05 µg/L. Furthermore, 28.0% of samples contained AFM1 in concentrations ranging from 0.005 to 0.007 µg/L in camel milk samples, and 18.0% of samples contained AFM1 in concentrations ranging from 0.005 to 0.007 µg/L in yak milk samples. Our survey study has expanded the current knowledge of the occurrence of AFM1 in milk from five dairy species in China, in particular the minor dairy species.

## 1. Introduction

Milk products are regarded as the consummate natural food for human beings, particularly during infancy [1]. The occurrence of aflatoxin M1 (AFM1) contamination in milk can potentially have critical health consequences for humans on a global scale. [2]. The concentrations of AFM1 that might be detected in milk are affected by a series of factors including dairy breed, geographic location and climate, and the direct proportion to aflatoxin B1 (AFB1) in feed ingested by dairy animals [3]. The sources of AFB1 contamination in the dairy animals’ feed often occurs in soybean meal, maize, wheat, bran, straw, and silage, and are very difficult to eradicate [4]. Hence, it is necessary to monitor the occurrence of AFM1 in dairy products.

The majority of dairy products are manufactured from cow milk, goat milk, buffalo milk, camel milk, and yak milk [5]. Assessing and monitoring the contamination of AFM1 in milk is one of the most effective approaches to providing data support as regards human exposure, and then to evaluate the health risks concerning the AFM1 intake. Moreover, it is also conducive to generalize the overall condition of AFM1 contamination, thus strengthening its monitoring and well-directed regulation [2].

Nowadays, although numerous studies in regards to AFM1 contamination in raw cow milk have been surveyed in the last few years in Tunisia [6], Morocco [7], Croatia [8], Serbia [9], Ethiopia [10], Tanzania [11], China [12], Pakistan [3], Qatar [13], Iran [14], Lebanon [15], Bangladesh [16], Ghana [17], and on around the world, the information that is available regarding AFM1 contamination in minor dairy species’ milk including goat milk, buffalo milk, camel milk, and yak milk is still fairly limited. The surveillance activity towards AFM1 contamination of cow milk should be strengthened and simultaneously extended to evaluate the milk coming from various minor dairy species. Here, the presence of AFM1 was determined using enzyme-linked immunosorbent assay method, with the detection limit 0.005 μg/L. This method is still a widely recognized standard screening method and generally applicated in routine analysis for AFM1 in milk [18]. Therefore, the objective of this study was to determine the AFM1 levels in milk from five dairy species in China, and also to better depict the safety of minor dairy species milk.

## 2. Results

### 2.1. AFM1 in Raw Cow Milk

For all 195 cow milk samples, 128 samples (65.7%) contained AFM1 above the detection limit of 0.005 µg/L with concentrations ranging from 0.005 to 0.191 µg/L (mean: 0.015 ± 0.022 µg/L, median is 0.008 µg/L), and 6 samples (3.1%) all collected from Sichuan province in October were contaminated with AFM1 content exceeding 0.05 µg/L (Table 1), which is the standard limit of EU.

### 2.2. AFM1 in Raw Goat Milk

For all 100 goat milk samples, 76.0% of samples contained AFM1 in concentrations ranging from 0.005 to 0.135 µg/L (mean: 0.022 ± 0.025 µg/L, median is 0.012 µg/L), and 9 samples (9.0%) all collected from Shanxi province in October were contaminated with AFM1 content exceeding 0.05 µg/L (Table 1).

### 2.3. AFM1 in Raw Buffalo Milk

For all 50 buffalo milk samples, 24 samples (48.0%) contained AFM1 above the detection limit in concentrations ranging from 0.005 to 0.089 µg/L (mean: 0.021 ± 0.022 µg/L, median is 0.009 µg/L), and 2 samples (4.0%) all collected in October were contaminated with AFM1 content higher than 0.05 µg/L (Table 1). For 25 samples sampled in May, 9 samples (36.0%) detected AFM1 ranging between 0.005 and 0.017 µg/L (mean: 0.010 ± 0.001 µg/L, median is 0.010 µg/L), and for the other 25 samples collected in October, AFM1 was positive in 15 samples (60.0%) from 0.005 to 0.089 µg/L (mean: 0.028 ± 0.026 µg/L, median is 0.009 µg/L) (Table 1).

### 2.4. AFM1 in Raw Camel Milk

For all 50 camel milk samples, 14 samples (28.0%) contained AFM1 above the detection limit in concentrations ranging from 0.005 to 0.007 µg/L (mean: 0.006 ± 0.001 µg/L, median is 0.006 µg/L), and no sample was above the AFM1 concentrations of 0.05 µg/L (Table 1). In May, AFM1 was positive in 8 to 25 samples (32.0%) ranging from 0.005 to 0.006 µg/L (mean: 0.006 ± 0.000 µg/L, median is 0.006 µg/L), and 6 to 25 samples (32.0%) contained AFM1 in the range of 0.005–0.007 µg/L in October (mean: 0.006 ± 0.001 µg/L, median is 0.006 µg/L) (Table 1).

### 2.5. AFM1 in Raw Yak Milk

For all 50 yak milk samples, 9 samples (18.0%) contained AFM1 above the detection limit in concentrations ranging from 0.005 to 0.007 µg/L (mean: 0.006 ± 0.000 µg/L, median is 0.006 µg/L), and no sample was contaminated with AFM1 content above 0.05 µg/L (Table 1). For 25 samples sampled in May, AFM1 was found in 8 samples (32.0%) with levels ranging from 0.005 to 0.007 µg/L (mean: 0.006 ± 0.000 µg/L, median is 0.005 µg/L), and one sample detected AFM1 with the content of 0.006 µg/L in the 25 samples collected in October (Table 1).

## 3. Discussion

**Figure 1:**
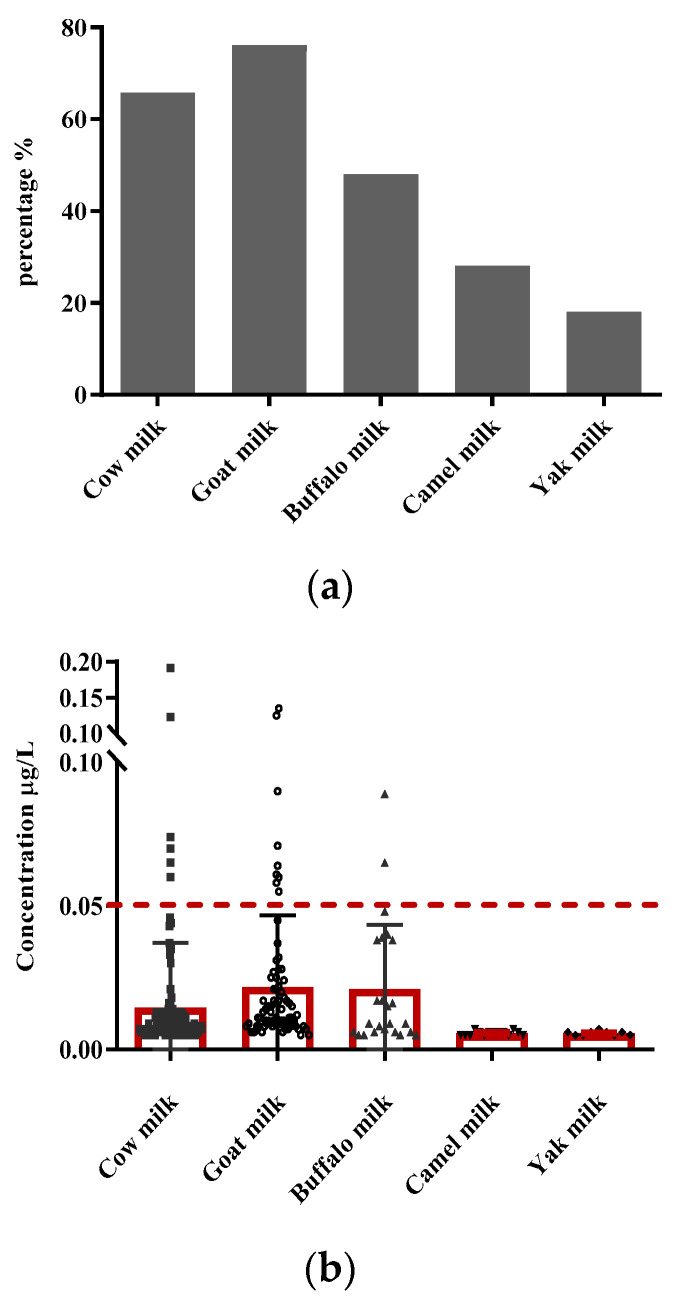
Percentage (**a**) and distribution (**b**) of positive samples to aflatoxin M1 in raw milk from five dairy species in China in 2016. Positive samples indicate an aflatoxin M1 concentration exceeding the quantification limit of 0.005 µg/L; percentage = (positive sample no)/(sample no) × 100%; Bars in distribution of positive samples (**b**) are plotted by mean with SD; 0.05 µg/L is the legal limit in the EU. The number of positive samples were 128, 76, 24, 14, and 9 in cow, goat, buffalo, camel, and yak milk, respectively. Of these, 6 cow milk, 9 goat milk, and 2 buffalo milk samples were contaminated with AFM1 above 0.05 µg/L.

The percentage and distribution of positive samples of AFM1 in raw cow, goat, buffalo, camel, and yak milk in China in 2016 are displayed in Figure 1. Contamination of AFM1 was positive in 65.7% of cow milk, 76.0% of goat milk, 48.0% of buffalo milk, 28.0% of camel milk, and 18.0% of yak milk. With respect to these positive samples, only 6 cow milk, 9 goat milk, and 2 buffalo milk samples do not meet the EU standard. The highest value of AFM1 levels is 0.191 µg/L in a cow milk sample, which is far below the American and Chinese limit standard. The review of recent published data on the AFM1 occurrence levels indicates that the global incidence of AFM1 in milk has remained one of the most critical and noteworthy food safety issues in developing regions of the world [19]. In China, the comprehensive application of quality and safety management in the dairy industry has effectively guaranteed the safety of raw milk and milk products [12]. Meanwhile, regular and uninterrupted surveillance of AFM1 in milk is necessary for continuously improving the safety of products [2].

**Figure 2:**
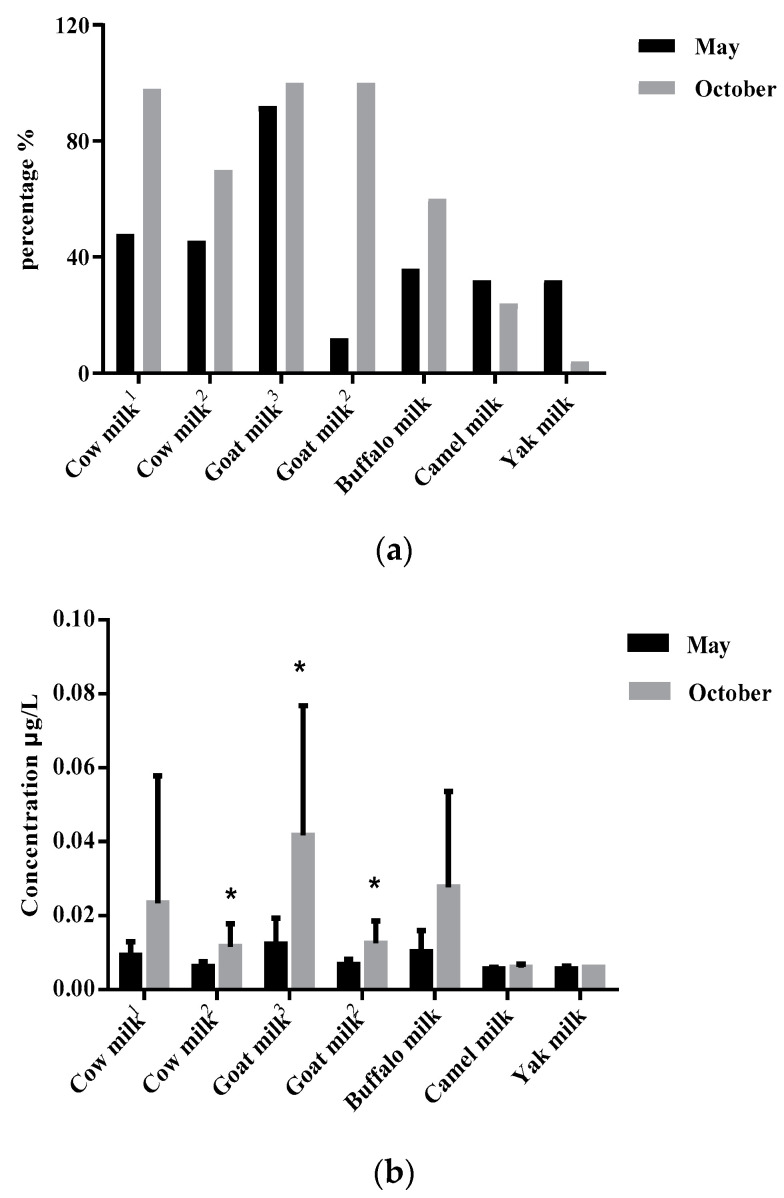
Comparisons of positive samples of aflatoxin M1 collected between May and October in raw milk from five dairy species in China showed by percentage (**a**) and mean (**b**). Positive samples indicate an aflatoxin M1 concentration exceeding the quantification limit of 0.005 µg/L; percentage = (positive sample no)/(sample no) × 100%; the symbol of * represents significant differences (*p* < 0.05) in aflatoxin M1 levels between May and October in each group; 1: samples collected from Sichuan; 2: samples collected from Shandong; 3: samples collected from Shanxi.

It is noteworthy that all of the AFM1 contaminated milk samples, which are exceeded the standard of EU, occurred in October. Furthermore, it showed that the percentage and mean of AFM1 in positive samples collected in October were all higher than those from May (Figure 2). The higher AFM1 contamination risk of milk samples is linked with the month of October because of its arid and cool climate. Silva et al. [20] indicated that the contamination levels of AFM1 in milk were much higher during autumn and winter seasons which might be due to the significant increase in feed intake when lactating cows are kept in arid and cool environments. In this process, if feed is contaminated with aflatoxin B1, this will give rise to increased AFM1 levels in raw milk [21]. Furthermore, a relatively limited amount natural pasture can be used in October. Compared with natural pasture, the risks of aflatoxin contamination in silage, maize, soybean meal, peanut meal, and other stored feed are much higher. Global studies have demonstrated that the high frequency of AFM1 contamination in milk poses a challenge in several countries possessing a dry and cool climate, including Brazil [22], China [12], Croatia [8], Egypt [23], Italy [24], Iran [25], Pakistan [26], and Serbia [27]. Hence, strict regulatory measures are necessary to control AFM1 contamination in milk below the EU limit standard especially in arid and cool climates; meanwhile, the application of good harvest practices, the standardization of storage and the lifespan of dairy species’ feed need to be conducted [2].

In China, there are plenty of dairy goats and the production of goat milk has already reached 290,457 tons in 2016 [28]. In the present study, 76.0% of goat milk samples were tested AFM1-positive, and 6.7% of them were over the EU limit. The incidence of AFM1 contamination in goat milk is a public sanitary security issue worldwide. A survey focus on the incidence of AFM1 in goat milk was performed in Lebanon, and all the samples were below the detection limit of the testing methods [29]. The concentrations of AFM1 in goat milk samples were in a range of 0.0056 to 0.0482 µg/L from Brazil [30], 0.00278 to 0.0408 µg/L from Croatia [31], 0.005 to 0.04 µg/L from Italy [32], 0.005 to 0.05 µg/L from Greece [33]. As aforementioned, all of the tested samples meet the standard of the EU. Moreover, AFM1 was detected in 31.6% of 60 goat milk samples in Iran, and 6.7% of the samples exceeded the EU limit [34]. In Syria, 63.7% of tested goat milk samples were contaminated with AFM1 ranging from 0.005 to 0.054 µg/L, and 9.1% of them exceeded the standard EU limit [35]. AFM1 contamination was positive in 33.3% of 150 goat milk samples in India, of which 10.0% were above the limit of EU [18]. The relatively high incidence and exceeding the standard rate of the EU limit of AFM1 in goat milk was reported by Pakistan [36], Egypt [37], Serbia [38], Jordan [39], and Nigeria [40].

Buffaloes are the second dairy species in milk production following dairy cows. China ranks third worldwide concerning the quantity of dairy buffalo herds and the production of buffalo milk [41]. Guo et al. [42] reported that AFM1 was detected in 62.5% of buffalo milk samples in south China, with ranges between 0.004 and 0.243 µg/L, and therein 5.9% of samples were over the EU limit. Subsequently, in this study, 48.0% of buffalo milk samples in China were determined AFM1-positive and only 4.0% of them were over the EU limit. The survey studies from Italy [24] and Turkey [43] on the occurrence of AFM1 in buffalo milk showed that 7.3% and 27.0% of the samples were, respectively, detected as AFM1-positive, and none of them were above the EU limit. The relatively high incidence and exceeding the standard rate of the EU limit of AFM1 in buffalo milk occurred in India [36], Iran [34,44], Pakistan [36,45], and Egypt [37].

In China, camel is mainly distributed in the grasslands and deserts of Xinjiang province. Commercial camel milk products can be consumed in local markets and have become more popular in recent years [46]. Our study indicates that the safety of camel milk is guaranteed in the aspect of AFM1 contamination in China. Similar results were also reported in Jordan [39], Nigeria [40], Qatar [47], and Iran [34], and the monitored samples were both within the EU permissible limit. However, another survey study from Iran states that 28.6% of camel milk contaminated with AFM1 was in excess of the maximum value specified in the EU limit [48]. Rates exceeding the EU standard were also revealed in Sudan (7.6%) and Egypt (20.0%) [37,49].

The yak is found throughout the Himalaya region, and over 95% of the global total yak population live in China at present [50]. The available information regarding the occurrence of AFM1 in yak milk is extremely limited. In this present study, the levels of AFM1 from all the yak milk samples in China were far below the level of 0.05 µg/L.

The differences detected with respect to AFM1 levels in milk samples, indicate that there is a relatively low risk of AFMI-contaminated dairy products derived from camel and yak milk. For cow, goat, and buffalo milk, the higher AFM1 contamination risk of milk samples were collected in October. The occurrence of AFM1 in milk samples is closely related to the contamination of feed [4]. Camels and yaks are usually raised on pasture, which offers the type of feed that is generally unsusceptible to AFMI contamination. The risk of aflatoxin contamination is much higher in silage, maize, soybean meal, peanut meal, and other stored feed. In October, natural pasture is relatively limited; therefore, the previous year’s silage that has been stored long-term, might be used. 

## 4. Conclusions

The percentage and distribution of raw milk from cow, goat, buffalo, camel, and yak in China in 2016 are summarized in this study. Of the cow milk samples, 65.7% contained AFM1 above the detection limit, and 3.1% of them above the EU limit. Of the goat milk samples, 76.0% were detected as AFM1-positive, and 9.0% of them above the EU limit. Of the buffalo milk samples, 48.0% were contaminated with AFM1 and contained 4.0%, exceeding the EU limit. Concerning camel milk and yak milk, none of the samples had AFM1 content exceeding the EU limit. If consumers ingest AFM1 from dairy products in high doses or over long periods, this will cause serious harm and health problems such as carcinogenicity, teratogenesis, genotoxicity, mutagenesis, and immunosuppression. Hence, considering the widespread aflatoxin contamination in some types of feed, the physicochemical and biological detoxification methods such as adsorbents, mold inhibitors, and atoxigenic biocompetitive strains should be applied to mitigate AFB1 contamination in susceptible feed. Moreover, the occurrence of AFM1 in milk samples needs to be regularly monitored, thus providing data regarding human exposure and potential health risks.

Furthermore, based on this survey and previous studies, we have summarized the overall situation of AFM1 contamination in minor dairy species milk in different countries (Table 2).

## 5. Materials and Methods

### 5.1. Sample Collection

In May and October 2016, the following samples were obtained: 195 raw cow milk samples from Sichuan and Shandong provinces, 100 raw goat milk samples from Shanxi and Shandong provinces, 50 raw buffalo milk samples from Guangxi province, 50 raw camel milk samples from Xinjiang province, and 50 raw yak milk samples from Guangxi province (Table 3). 100 mL of each sample was stored at −20 °C until analysis was conducted.

### 5.2. Determination of AFM1 Levels

Sample test: The quantitative determination of AFM1 in samples was detected using RIDASCREEN^®^ Aflatoxin M1 test kit (R1111, R-Biopharm AG, Darmstadt, Germany), with the detection limit 0.005 μg/L. This method is still a widely recognized standard screening method, and generally applicated in routine analysis for AFM1 in milk [18]. The 8 mL sample of milk was degreased by centrifugation at 3500 g and 10 °C for 10 min to remove milk fat through discarding the upper creamy layer, and 100 µL of the defatted supernatant was subjected to the test. The test was performed in accordance with the operating instructions attached to the kit. All raw milk samples were determinated in duplicate. Concentrations of AFM1 in samples were calculated from their absorbance value and standard curve that was obtained from AFM1 standard solutions (0, 0.005, 0.01, 0.02, 0.04, and 0.08 µg/L) in the kit although the standard curve could calculate the minimum concentration of 0.003 µg/L and maximum concentration of 0.1 µg/L. The quantification limit for this method ranged from 0.005 to 0.08 μg/L, in reference to the operating instructions and a previous study [1]. A sample was considered to be negative for AFM1 if the levels were below the minimum detection limit of 0.005 µg/L. Moreover, a sample with AFM1 concentration greater than 0.08 μg/L was diluted with sample diluent solution in the kit and tested again.

Quality control: Control samples (standard solutions: 0, 0.005, 0.01, 0.02, 0.04, and 0.08 µg/L in the kit) were added in each type of the raw milk samples that have been tested below the detection limit (control negative for AFM1). They were adjusted to 0.01, 0.03, 0.05, and 0.08 µg/L AFM1 to test the recovery and RSD (relative standard deviation) values in order to validate their accuracy. The number of control milk samples per spiked concentration was twelve. Generally, recovery of AFM1 in raw milk ranged from 80.0 to 120.0%, while RSD values of less than 10% were considered acceptable. The consequences of the quality control in this study are presented in Table 4.

### 5.3. Statistical Analysis

Differences in five species of raw milk were statistically analyzed by non-parametric factorial Kruskal-Wallis sum-rank test, using SPSS Statistics19.0 (SPSS, Inc., Chicago, IL, USA). Furthermore, differences in period, province, and different species of raw milk in the same province were statistically analyzed by non-parametric factorial Mann-Whitney U test, using SPSS Statistics19.0 (SPSS, Inc., Chicago, IL, USA).

## Figures and Tables

**Table 1:** Aflatoxin M1 (AFM1) positivity/concentration in raw milk from five dairy species in China in 2016.

Raw Milk	Province	Period	Sample No.	Positive Sample No.	Mean ± SD (µg/L)	Median (µg/L)	Concentration Range (µg/L)	No. of Samples with AFM1 Levels > 0.05 µg/L
Cowmilk	Sichuan	May	50	24 (48.0%)	0.009 ± 0.004	0.009	0.005–0.018	0
		October	49	48 (98.0%)	0.023 ± 0.034	0.008	0.005–0.191	6 (12.2%)
		Total	99	72 (72.7%)	0.019 ± 0.029	0.008	0.005–0.191	6 (6.1%)
	Shandong	May	46	21 (45.7%)	0.006 ± 0.001	0.006	0.005–0.010	0
		October	50	35 (70.0%)	0.012 ±0.006	0.011	0.005–0.043	0
		Total	96	56 (58.3%)	0.010 ± 0.006	0.009	0.005–0.043	0
	*Total Cow milk*		195	128 (65.7%)	0.015 ± 0.022 ^A^	0.008	0.005–0.191	6 (3.1%)
Goatmilk	Shanxi	May	25	23 (92.0%)	0.012 ± 0.006	0.010	0.005–0.028	0
		October	25	25 (100%)	0.042 ± 0.035	0.028	0.006–0.135	9 (36.0%)
		Total	50	48 (96.0%)	0.028 ± 0.030	0.016	0.005–0.135	9 (18.0%)
	Shandong	May	25	3 (12.0%)	0.007 ± 0.002	0.007	0.005–0.008	0
		October	25	25 (100%)	0.013 ± 0.006	0.011	0.007–0.037	0
		Total	50	28 (56.0%)	0.012 ± 0.006	0.010	0.005–0.037	0
	*Total Goat milk*		100	76 (76.0%)	0.022 ± 0.025 ^B^	0.012	0.005–0.135	9 (9.0%)
Buffalomilk	Guangxi	May	25	9 (36.0%)	0.010 ± 0.001	0.010	0.005–0.017	0
		October	25	15 (60.0%)	0.028 ± 0.026	0.009	0.005–0.089	2 (8.0%)
	*Total Buffalo milk*		50	24 (48.0%)	0.021 ± 0.022 ^AB^	0.009	0.005–0.089	2 (4.0%)
Camelmilk	Xinjiang	May	25	8 (32.0%)	0.006 ± 0.000	0.006	0.005–0.006	0
		October	25	6 (24.0%)	0.006 ± 0.001	0.006	0.005–0.007	0
	*Total Camel milk*		50	14 (28.0%)	0.006 ± 0.001 ^C^	0.006	0.005–0.007	0
Yakmilk	Sichuan	May	25	8 (32.0%)	0.006 ± 0.000	0.005	0.005–0.007	0
		October	25	1 (4.0%)	0.006 ± 0.001	0.006	0.006	0
	*Total Yak milk*		50	9 (18.0%)	0.006 ± 0.000 ^C^	0.006	0.005–0.007	0

Positive samples indicate an aflatoxin M1 concentration exceeding the quantification limit of 0.005 µg/L. The letters A, B, and C represent significant differences (*p* < 0.05) in aflatoxin M1 levels between cow milk, goat milk, buffalo milk, camel milk, and yak milk, respectively.

**Table 2:** Frequency of AFM1 contamination in minor dairy species milk samples in different countries.

Raw Milk	Country	Sample No.	Positive Sample No.	Concentration Range (µg/L)	No. of Samples with AFM1 Levels > 0.05 µg/L	References
Goat milk	Lebanon	3	0	a	0	[29]
	Brazil	108	108 (100%)	0.0056–0.0482	0	[30]
	Croatia	32	30 (93.8%)	0.00278–0.00408	0	[31]
	Italy	208	36 (17.3%)	0.005–0.04	0	[32]
	Greece	22	12 (54.6%)	0.005–<0.05	0	[33]
	Iran	60	19 (31.6%)	0.005–>0.05	4 (6.7%)	[34]
	China	100	76 (76.0%)	0.005–0.135	9 (9.0%)	This study
	Syria	11	7 (63.7%)	0.005–0.054	1 (9.1%)	[35]
	India	150	50 (33.3%)	0.005–>0.05	5 (10.0%)	[18]
	Pakistan	62	26 (42.0%)	0.008–0.1405	8 (13.0%)	[36]
	Egypt	50	18 (63.0%)	0.01–0.25	13 (26.0%)	[37]
	Serbia	10	8 (80.0%)	0.008–0.24	4 (40.0%)	[38]
	Jordan	20	20 (100%)	0.002025–0.12589	15 (75.0%)	[39]
	Nigeria	87	43 (49.5%)	LOD-3.108	24 (27.6%)	[40]
Buffalo milk	Italy	388	28 (7.3%)	0.004–<0.05	0	[24]
	Turkey	126	34 (27.0%)	0.008–0.032	0	[43]
	China	136	85 (62.5%)	0.004–0.243	8 (5.9%)	[42]
	China	50	24 (48.0%)	0.005–0.089	2 (4.0%)	This study
	India	150	50 (33.3%)	0.005–>0.05	5 (10.0%)	[18]
	Iran	75	29 (38.7%)	0.005–>0.05	6 (8.0%)	[34]
	Iran	60	46 (76.7%)	0.0127–0.4227	32 (53.4%)	[44]
	Pakistan	94	46 (49.0%)	0.004–0.350	16 (17.1%)	[45]
	Pakistan	76	43 (56.6%)	0.008–0.3205	16 (21.1%)	[36]
	Egypt	50	32 (64.0%)	0.01–>0.25	24 (48.0%)	[37]
Camel milk	Jordan	10	10 (100%)	0.002357–0.009652	0	[39]
	Nigeria	25	0	a	0	[40]
	China	50	14 (28.0%)	0.005–0.007	0	This study
	Qatar	26	12 (46.2%)	NA	0	[47]
	Sudan	66	5 (7.6%)	0.05–0.1	5 (7.6%)	[49]
	Iran	40	5 (12.5%)	0.005–<0.05	0	[34]
	Iran	70	NA	0.00519–0.015017	20 (28.6%)	[48]
	Egypt	25	9 (36.0%)	0.01–<0.25	5 (20.0%)	[37]
Yak milk	China	50	9 (18.0%)	0.005–0.007	0	This study

Positive samples indicate an aflatoxin M1 concentration exceeding the quantification limit of the corresponding reference. a: below detection limit. LOD: limit of detection. NA: not mentioned.

**Table 3:** Sample characterization of five dairy species milk collected in China in this study.

Raw Milk	Provinces	Sample No.	
		May	October
Cow milk	Sichuan	50	49
	Shandong	46	50
Goat milk	Shanxi	25	25
	Shandong	25	25
Buffalo milk	Guangxi	25	25
Camel milk	Xinjiang	25	25
Yak milk	Sichuan	25	25

**Table 4:** Recovery and RSD of aflatoxin M1 from negative control milk samples spiked with known concentrations of aflatoxin M1.

AFM1 Spiked (μg/L)	AFM1 Found (μg/L)	Recovery (%)	RSD (%)
0.01	0.008–0.012	80–120	2.4–9.1
0.03	0.027–0.036	90–120	3.5–7.6
0.05	0.045–0.059	90–118	4.2–8.1
0.08	0.070–0.092	87.5–115	2.6–7.8

## Data Availability

Not applicable.
